# Increase in ACC GABA+ levels correlate with decrease in migraine frequency, intensity and disability over time

**DOI:** 10.1186/s10194-021-01352-1

**Published:** 2021-12-13

**Authors:** Aimie L. Peek, Andrew M. Leaver, Sheryl Foster, Nicolaas A. Puts, Georg Oeltzschner, Luke Henderson, Graham Galloway, Karl Ng, Kathryn Refshauge, Trudy Rebbeck

**Affiliations:** 1 Faculty of Medicine and Health, University of Sydney, Camperdown, New South Wales 2141 Australia; 2 NHMRC Centre of Research Excellence in Road Traffic Injury Recovery, Brisbane, Queensland Australia; 3 Department of Radiology, Westmead Hospital, Hawkesbury Road, Westmead, New South Wales 2145 Australia; 4 Department of Forensic and Neurodevelopmental Sciences, Sackler Institute for Translational Neurodevelopment, Institute of Psychiatry, Psychology, and Neuroscience, Kings College London, London, UK; 5 Department of Radiology and Radiological Science, Johns Hopkins University School of Medicine, Baltimore, MD 21287 USA; 6 F.M. Kirby Research Center for Functional Brain Imaging, Kennedy Krieger Institute, Baltimore, MD 21205 USA; 7 School of Medical Sciences, Brain and Mind Centre, University of Sydney, Camperdown, Australia; 8 The University of Queensland, St Lucia, Queensland 4072 Australia; 9 Translational Research Institute, 37 Kent Street, Woolloongabba, Queensland 4102 Australia; 10 Department of Neurology, Royal North Shore Hospital, Reserve Road, St Leonards, New South Wales 2065 Australia

**Keywords:** GABA, MRS, Migraine, Anti-CGRP, Pain, Longitudinal

## Abstract

**Background:**

An imbalance between inhibitory and excitatory neurometabolites has been implicated in chronic pain. Prior work identified elevated levels of Gamma-aminobutyric acid + macromolecules (“GABA+”) using magnetic resonance spectroscopy (MRS) in people with migraine. What is not understood is whether this increase in GABA+ is a cause, or consequence of living with, chronic migraine. Therefore, to further elucidate the nature of the elevated GABA+ levels reported in migraine, this study aimed to observe how GABA+ levels change in response to changes in the clinical characteristics of migraine over time.

**Methods:**

We observed people with chronic migraine (ICHD-3) over 3-months as their treatment was escalated in line with the Australian Pharmaceutical Benefits Scheme (PBS). Participants underwent an MRS scan and completed questionnaires regarding migraine frequency, intensity (HIT-6) and disability (WHODAS) at baseline and following the routine 3 months treatment escalation to provide the potential for some participants to recover. We were therefore able to monitor changes in brain neurochemistry as clinical characteristics potentially changed over time.

**Results:**

The results, from 18 participants who completed both baseline and follow-up measures, demonstrated that improvements in migraine frequency, intensity and disability were associated with an increase in GABA+ levels in the anterior cingulate cortex (ACC); migraine frequency (*r* = − 0.51, *p* = 0.03), intensity (*r* = − 0.51, p = 0.03) and disability (*r* = − 0.53, *p* = 0.02). However, this was not seen in the posterior cingulate gyrus (PCG). An incidental observation found those who happened to have their treatment escalated with CGRP-monoclonal antibodies (CGRP-mAbs) (*n* = 10) had a greater increase in ACC GABA+ levels (mean difference 0.54 IU IQR [0.02 to 1.05], *p* = 0.05) and reduction in migraine frequency (mean difference 10.3 IQR [2.52 to 18.07], *p* = 0.01) compared to those who did not (*n* = 8).

**Conclusion:**

The correlation between an increase in ACC GABA+ levels with improvement in clinical characteristics of migraine, suggest previously reported elevated GABA+ levels may not be a cause of migraine, but a protective mechanism attempting to suppress further migraine attacks.

**Supplementary Information:**

The online version contains supplementary material available at 10.1186/s10194-021-01352-1.

## Background

Migraine is the leading neurological cause of disability worldwide [1], with an estimated global prevalence of 14.7% [2]. Chronic migraine is defined by the International Classification of Headache disorders (ICHD-3) as headaches that persist for more than or equal to 15 days a month, with at least 8 of those days having features of migraine and persisting for at least 3-months [3]. Despite chronic migraine only representing 7.7% of the entire migraine population [4], compared to episodic migraine it is associated with higher healthcare utilization, work disability, and reduction in quality of life [5–7] . Even though recent treatments are showing better effects, the responsiveness rate remains less than 50%, leaving 50% of people with ongoing symptoms of chronic migraine [8, 9]. Treatment development is hindered by the limited understanding of the pathophysiology of chronic migraine. Given this, there is a global call to understand the mechanism of chronic migraine to enable the development of more effective treatment strategies [10].

Chronic migraine has been attributed to several proposed mechanisms involving both peripheral and central mediators. One proposed mechanism of migraine is an imbalance between the main inhibitory and excitatory neurometabolites, gamma-aminobutyric acid (GABA) and glutamate. Studying these metabolites has previously proved challenging owing to spectral overlap of more abundant neurometabolites at clinical magnetic resonance imaging (MRI) field strength [11]. However, Advanced ^1^H-Magnetic Resonance Spectroscopy (MRS) techniques such as MEGA-PRESS [12] address these limitations and allow for more reliable quantification of GABA or GABA+ co-edited macromolecules (GABA+) whilst also reporting the composite glutamate-glutamine-glutathione (Glx).

Several GABA/ GABA+ optimized cross-sectional MRS studies have investigated the imbalance of inhibition and excitation as a potential underlying cause of chronic migraine [13–16]. Our recent meta-analysis pooled results from 5 studies that reported levels of GABA and GABA+ in the anterior cingulate cortex (ACC), insula, occipital lobe and posterior cingulate cortex (PCG) and 6 studies that reported levels of Glx in the ACC, occipital lobe, PCG and thalamus of people with migraine [17]. We found GABA or GABA+ levels were significantly elevated in individuals with migraine compared to controls, yet there was no difference in Glx levels. These results challenge the concept of loss of inhibition leading to cortical hyperexcitability, where reduced levels of GABA compared to controls might be anticipated [18]. We might postulate that directional differences in GABA levels may be dependent on whether GABA is working within inhibitory or facilitatory circuits in the region being studied, which may result in either decreased or increased axonal firing respectively [19]. Nevertheless, this somewhat unexpected increase in GABA suggests a more complex relationship between the inhibitory and excitatory neurometabolites involved in migraine and warrants further investigation.

In investigating the role of neurometabolites in migraine, the region of brain to be examined must be considered. Studies of the PCG have demonstrated both elevated levels of GABA+ [14, 16] and an association between elevated GABA+ levels and central sensitization [20] in people with migraine. Another region, the ACC, has a well-established role in pain processing and modulation [21], and changes in ACC GABA+ levels have been reported in pain conditions such as fibromyalgia [22] and pelvic pain [23].

The elevated baseline levels of GABA+ observed in this cohort [24] and by others [14, 20, 25] have been proposed as a potential cause of migraine, due to being present in people with migraine but not in healthy controls. However, given the cross-sectional nature of the studies, the temporal and directional nature of these findings are unknown. Therefore, to further elucidate the nature of the elevated GABA+ levels in people with migraine, longitudinal studies, that examine the association between *change* in migraine characteristics (e.g. migraine frequency, pain intensity and disability) and *change* in GABA+ or Glx levels are required. Examining a cohort before and after treatment that is known to have a reasonable response rate (e.g. Onabotulinumtoxin A or calcitonin gene-related peptide monoclonal antibodies (CGRP-mAbs); response rate ~ 40% [8, 9], allows such an opportunity.

### Aims

Our primary aim was to determine whether there is an association between *change* in GABA+ levels and *change* in migraine characteristics over time to further elucidate the nature of GABA’s role in migraine. Secondary aims were to determine whether there is an association between baseline neurometabolite levels and change in clinical characteristics and / or change in neurometabolite levels. This would establish whether baseline levels of GABA+ could predict change either clinically or neurochemically.

## Methods

### Study design

This study was a longitudinal cohort study, observing a group of migraine participants who formed part of a larger cross-sectional multi-group study [16].

### Participants

There were 20 participants with chronic migraine with or without aura as diagnosed by the ICHD-3 [3] recruited by a neurologist (KN) working in private practice (4 males, 16 females, mean age 39.7 ± 10 years). To be eligible to receive treatment (Botox® or CGRP- mAbs) under the Australian Pharmaceutical Benefits Scheme (PBS), participants were required to have experienced an average of at least 15 headache days a month, (8 days of which migrainous) for over 6-months and having failed three or more prophylactic migraine medications [26]. In addition, they were required to have at least moderate headache related disability measured as a HIT-6 score [27] exceeding 50 at the time of recruitment. All participants were recruited when they were due to start a new treatment regimen based on escalating care in line with the PBS guidelines [26]. Participants were included if they received any peripherally acting evidence-based medication to escalate their care (e.g. Onabotulinumtoxin A, CGRP-monoclonal antibodies (CGRP-mAbs). Treatment could be escalated with either Onabotulinumtoxin A injections 155 mg administered 12 weekly according to the PREEMPT protocol [8] or CGRP-mAbs as erenumab 70 to 140 mg self-administered by injection monthly.

Participants were excluded if they were taking medication known to affect GABA levels at baseline (e.g. diazepam, topiramate or gabapentin), had contraindications to MRI (e.g. claustrophobia, MR-unsafe devices/implants) or conditions that compromised MR spectroscopy (e.g. metal braces). In addition, participants were excluded if they experienced any acute health complaints in the 5 days prior to the scan, were diagnosed with a psychiatric or neurological condition, experienced pain in other regions of the body, or if they were unable to communicate in the English language.

To test the reliability of collecting longitudinal MRS, 5 healthy participants (2 males, 3 females, mean age 44.8 ± 10.0 years) were recruited for the study through advertisements placed on university and hospital noticeboards. These participants had no history of chronic pain, headache or health conditions and had no contraindications of MRI.

### Procedure

Potential participants were identified by the treating neurologist, who provided study information, and gained consent to be contacted by the research team. The research team screened potential participants for eligibility by telephone, further explained the study and gained written informed consent. Participants immediately started an online pain diary, completed initial questionnaires of headache severity (Headache Impact Test- HIT-6), disability (World Health Organization Disability Assessment Schedule-WHODAS 2.0–12), pain sensitivity (Central Sensitisation Inventory-CSI) and psychological wellbeing (Depression Anxiety and Stress Scale- DASS-21). Questionnaires were completed using the online platform, REsearch Data Capture® (REDcap) [28]. Participants were scanned in their interictal phase and asked to refrain from taking pain medication, caffeine, nicotine or alcohol on the day of the MRI/MRS scan. Following the initial scan participants started their new treatment regimen. At 3-month follow-up participants repeated headache pain and disability questionnaires and attended for a follow-up MRI/MRS scan under the same conditions as their first scan.

### Clinical outcome measures

Validated patient reported outcome measures were chosen to evaluate change in headache severity and disability over time. The HIT-6 was chosen due to being specifically designed and validated as a measure of adverse headache impact in both clinical practice and research [27, 29]. Scores range from 36 to 78 with higher scores corresponding to higher levels of disability. The WHODAS 2.0–12 was chosen as a global measure of disability due to its reliability and sensitivity to detecting change in disability over time [30]. Scores range from 12 to 60, with 60 indicating the highest level of disability.

Headache frequency was measured through an online weekly pain diary. Participants recorded the number of days they experienced migraine each week and rated the migraine severity using the Numerical Rating Scale (NRS) where 0 was no pain, and 100 the worst imaginable pain.

### Neurometabolites of interest

The primary aim of the study was to understand the role of GABA. We therefore focused on GABA+ levels, as this currently reflects the most reliable method to report GABA when using a repeat measure design. The Glx composite signal was a secondary target, representing glutamate with additional contributions from glutamine (Gln) and glutathione (GSH). The composite signal is reported since Glu, Gln and GSH are heavily overlapped at 3 T field strength and therefore difficult to resolve from each other reliably [31].

### MRI/MRS data acquisition

**Fig. 1:**
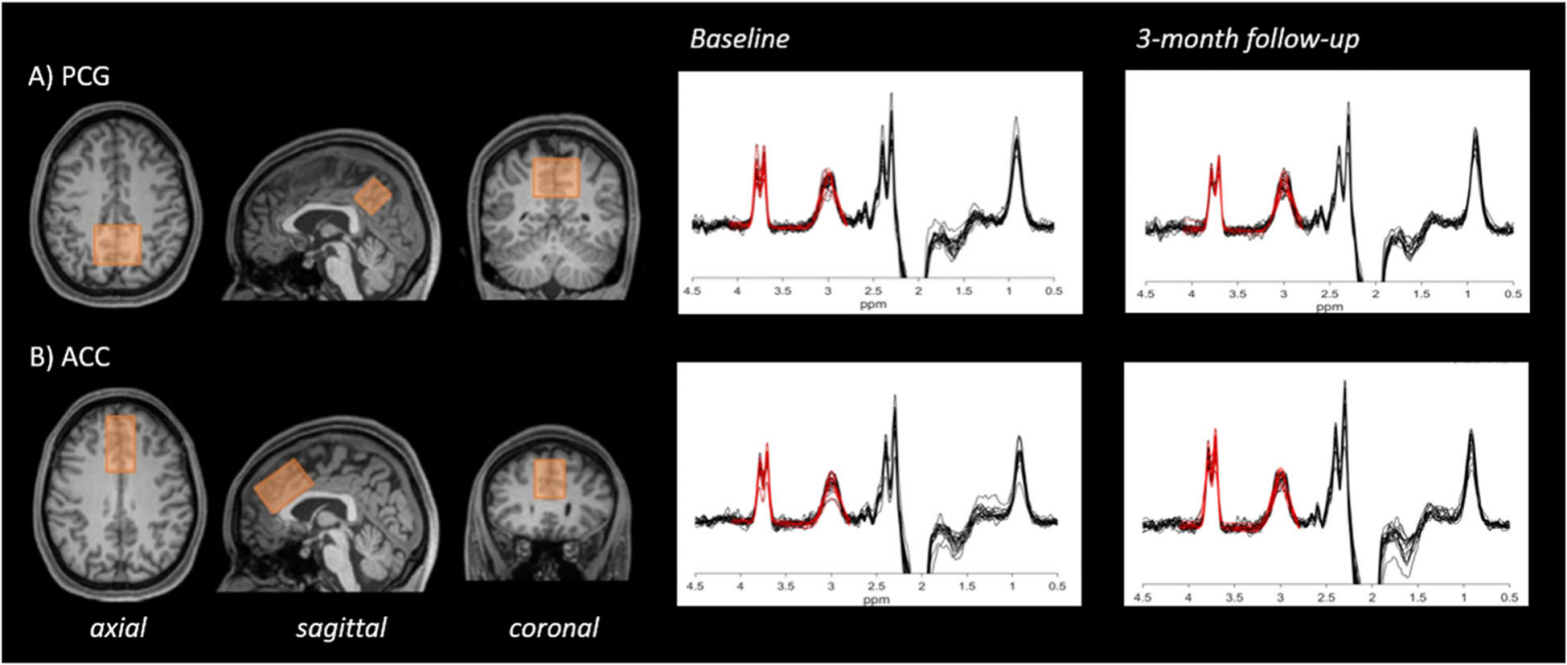
Exemplary voxel placement and an overlay of all spectra from A) PCG and B) ACC at baseline and 3-month follow-up

All participants were scanned on a Siemens 3 T Magnetom Prisma (Erlangen, Germany) with 64-channel head coil. High resolution 3D T_1_-weighted structural images (repetition time (TR) = 2400 ms; echo time (TE) = 2.21 ms; inversion time (TI) = 1150 ms; voxel size = 1.0 × 1.0 × 1.0 mm^3^; FOV = 256 mm; matrix = 256 × 256; acquisition time = 4 min 35 s) were acquired to inform voxel placement (previously described [24]) and for use in tissue segmentation. A 2D T_2_-weighted series (TR = 7490 ms; TE = 99 ms; voxel size = 0.6 × 0.3 mm^3^; FOV = 220 mm; matrix = 384 × 288; acquisition time = 2 min 24 s) was also acquired and sent to a consultant radiologist to review and report any incidental finding. MRS data were acquired using the MEGA-PRESS sequence from two regions shown in Fig. 1, the posterior cingulate gyrus (PCG, voxel size 25 (AP) × 40 (RL) × 25 (CC) mm ^3^) and the anterior cingulate cortex (ACC, 40 (AP) × 25 (RL) × 25 (CC) mm^3^). Common parameters for both voxels were: TR = 2000 ms; TE = 68 ms; 192 averages (96 ON, 96 OFF); 2048 data points; spectral width = 2000 Hz; editing pulse frequencies set to 1.9 ppm and 7.5 ppm for editing of GABA+; editing pulse bandwidth = 70 Hz. Water-unsuppressed MEGA-PRESS data (with water suppression RF pulses deactivated) were also acquired from each voxel to perform eddy-current correction and water-scaled quantification.

### MRS data processing

MRS data were processed using the open-source MATLAB-based analysis toolbox Gannet 3.1 [32], including data loading, coil combination, frequency-and-phase-correction of individual transients using the Spectral Registration algorithm [33], and averaging. The 3-ppm GABA+ and 3.75-ppm Glx signals in the difference spectrum were modelled with a single Gaussian and a dual-Lorentzian peak, respectively, including terms for the baseline slope between the two signals. The water signal in the water reference spectrum was modelled with a single Voigtian peak. The voxels were co-registered to the T_1_-weighted structural acquisition, which was segmented using built-in SPM12 functions. GABA+ and Glx levels were quantified relative to the internal tissue water signal, accounting for tissue composition of the voxel, as well as different water content and relaxation times for grey matter, white matter, and cerebrospinal fluid. Alpha-corrected GABA concentration estimates were reported, accounting for the fact that GABA+ and Glx concentrations differ between grey and white matter at a ratio of ~ 2:1 [34].

### Spectroscopy quality

Spectra were visually examined for artefacts by two investigators with eight and ten years’ experience of spectral editing (GO, NP). Spectra were excluded if they demonstrated significant motion artefact or insufficient water suppression (*n* = 1, healthy participant, ACC voxel). All remaining spectra achieved a fit error below the recommended quality threshold of 10% [32]. The mean fit error was 4.61 ± 0.95% in the PCG and 4.57 ± 0.67% in the ACC.

### MRS test-retest reliability

The test-retest reliability of the MEGA-PRESS acquisition over the same 3-month time period was determined in the 5 healthy control participants. One ACC acquisition was excluded from the analysis due to substantial motion artefact. Results demonstrated a test-retest coefficient of variance (CV) of 10% in PCG, and 12.6% in the ACC. This is in agreement with previous MEGA-PRESS studies of GABA+ [35–37] and provides evidence of the reliability of the MRS collection in this longitudinal investigation.

### Statistical analysis

The power calculation was based on our previous work [14]. A sample size of *n* = 17 was required to detect a 0.2 IU change in GABA+ level with 80% power and therefore, 20 participants were recruited to allow for a 15% dropout rate.

Participants’ baseline characteristics were reported using descriptive statistics, mean and standard deviation for normally distributed data, and median interquartile range for non-normally distributed data.

The outcomes of GABA+ levels, Glx levels, migraine days per month, HIT-6 scores and WHODAS scores were included in the statistical analysis. Normality for each variable was assessed using Kolmogorov-Smirnov and Shapiro-Wilk tests. Paired sample t-tests were used to examine change in clinical characteristics (migraine days, HIT-6, WHODAS) and change in neurometabolite levels (GABA+, Glx) from baseline to 3-month follow-up.

In addressing our primary aim to determine the degree of correlation between *change* in characteristics of migraine and *change* in neurometabolite levels, and to determine the correlation between *baseline* and *change* in neurometabolite levels, Pearson’s (*r*) correlation was used- due to the normal distribution of data. Correlations *r* > 0.07 were considered strong, 0.4 to 0.69 moderate, 0.1–0.39 weak, < 0.1 negligible [38, 39].

Neurometabolite levels and clinical outcome measures were plotted as raincloud plots, which provide a transparent method of data visualisation [40]. Raincloud plots consist of a half-violin plot to visualize the distribution, a box plot to highlight the median and 95% confidence intervals, and scatter plots with lines connecting each individual participants score at baseline and 3-month follow-up.

Post-hoc testing was carried out to explore if *change* in GABA+ or Glx level was different in those who were escalated with CGRP-mAbs treatment compared to Onabotulinumtoxin A. Due to the normal distribution of data an independent sample t-test was used to determine any between-group differences. A point biserial correlation (Pearson’s (*r*) correlation using one dichotomous variable and one continuous variable) was then used to determine correlations between group (CGRP-mAbs Yes/No) with headache frequency, intensity and disability.

An alpha level of 0.05 was used for all statistical tests. Statistical testing was carried out using SPSS version 26 [41] and data visualisation was performed in R version 4.0.2 [42].

## Results

Of the 20 participants recruited, 18 were included in the final analysis. One was excluded following MRS because they had taken diazepam prior to being scanned, thus not meeting the study’s inclusion criteria, and one did not complete their follow-up scan. Of the final 18 participants, 8 had been escalated to Onabotulinumtoxin A, and 10 with CGRP-mAbs. All participants had received two doses of medication between baseline and 3-month follow-up.

### Participants

#### Baseline characteristics of study population

The mean ± SD duration of migraine was 21 ± 11.0 years in the participants included in the final analysis (*n* = 18). Participants experienced on average 16.7 ± 5.1 headache days in the month preceding the baseline scan, had an average pain intensity of 66.1% ± 22.9 in the week preceding the scan (Table 1). Participants were scanned in the interictal phase, however due to the chronic nature of the symptoms some still had residual head pain as reflected in the time since migraine (Table 1). Overall, the group’s baseline psychological status indicated that on average, participants were mildly depressed and had moderate anxiety and stress levels [43].

**Table 1:** Clinical characteristics of participants

	Migraine (n = 18)
**Demographics** (mean ± SD)	
Age	39.0 ± 10.0
Female (n, %)	15 (79%)
BMI	26.8 ± 6.2
Educational level (University educated n, %)	12 (63.1%)
**Migraine Characteristics** (mean ± SD)	
Duration (years)	21.0 ± 11.0
Aura (n, %)	12.0 (66.7%)
Pain intensity in last week (NRS %)	66.1 ± 22.9
Escalated with CGRP-mAbs (n, %)	10.0 (55.6%)
**Psychological Status** (mean ± SD)	
DASS total score (range: 0 to 126)	21.6.0 ± 14.0
- Depression (range: 0 to 42)	4.4 ± 5.7
- Anxiety (range: 0 to 42)	6.0 ± 5.4
- Stress (range: 0 to 42)	11.2 ± 6.1
**Symptoms** (median (IQR))	
Pain at time of 1st scan [NRS %]	40.0 (10.0 to 60.0)
Pain at time of 2nd scan [NRS %]	10.5 (0.0 to 50.0)
Time since migraine 1st scan [hours]	11.0 (0.0 to 48.0)
Time since migraine 2nd scan [hours]	36.0 (4.0 to 168.0)
Central Sensitisation Inventory**˄** (range: 0 to 100)	45.0 (36.0 to 50.0)
**Baseline Clinical status** (mean ± SD)	
Migraine frequency (days per month)	16.7 ± 5.1
WHODAS 2.0 (range: 12 to 60)	24.8 ± 16.1
HIT-6 **ǂ** (range: 36 to 78)	65.7 ± 6.6
GABA+ ACC [institutional units, IU]	4.51 ± 0.38
GABA+ PCG [IU]	4.93 ± 0.62
Glx ACC [IU]	13.51 ± 1.25
Glx PCG [IU]	12.90 ± 1.86

Clinical characteristics of participants included in final analysis (*n* = 18). Data reported as mean ± SD, except where stated non-normally distributed data is reported as Median (IQR). For DASS, CSI, WHODAS, HIT-6 a higher score indicates greater infliction. ˄CSI > 40 indicates central sensitization, ǂHit-6 score > 60 indicate severe impact.

### Clinical changes from baseline to 3-month follow-up

In order to address our primary aim, we first report observed changes in migraine characteristics and brain neurometabolite levels from baseline to 3-month follow-up.

#### Change in migraine characteristics from baseline to 3-month follow-up

**Fig. 2:**
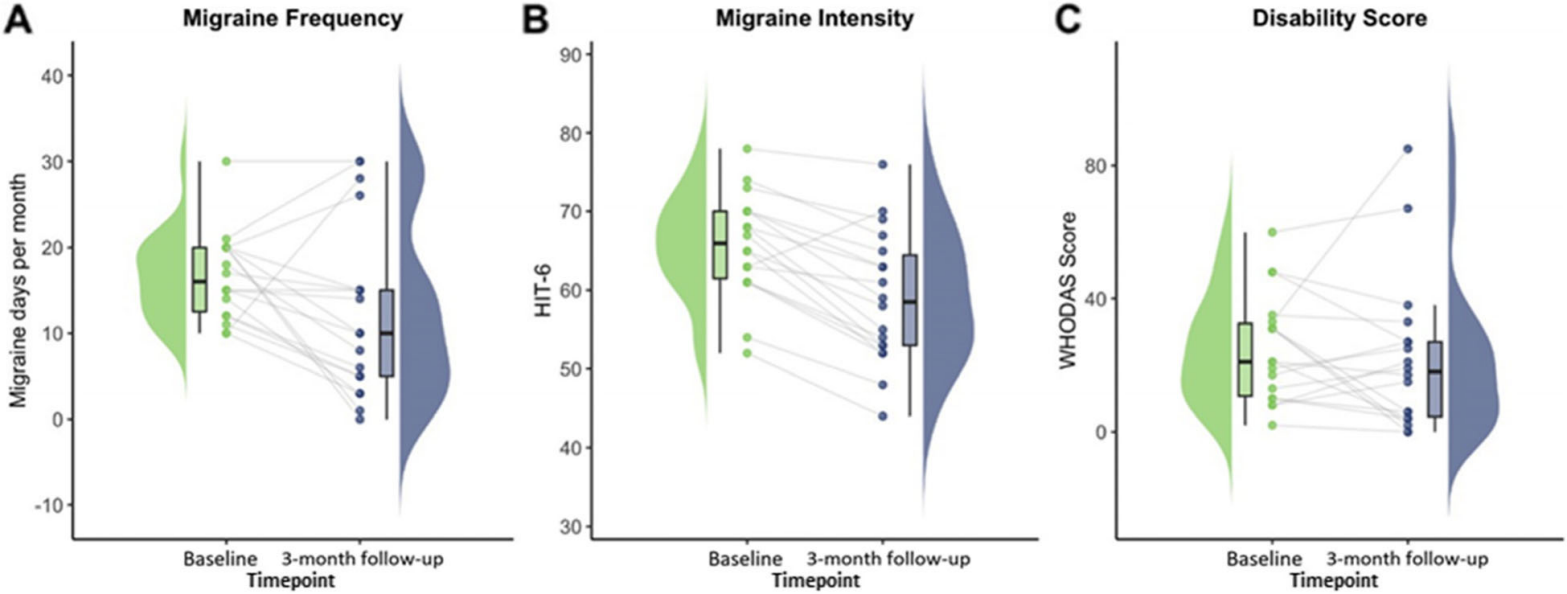
Change in migraine characteristics from baseline to 3-month follow-up. Raincloud plots for change in A) migraine frequency (migraine days per month), B) migraine intensity (HIT-6 score) and C) disability (WHODAS score). Each individual plot represents change in clinical characteristics from baseline to 3-month follow-up in participants with migraine (*n* = 18). Green data points represent migraine characteristics at baseline and blue at 3-month follow-up. The grey lines represent change in individual participants scores over time

Overall the group experienced an improvement in clinical characteristics of migraine from baseline to 3-month follow-up. The mean ± SD headache frequency was 16.7 ± 5.1 days per month at baseline and 12.4 ± 10.0 days at 3-month follow-up (mean difference − 4.22 days, 95% CI [− 8.78 to 0.33] days, *t* (17) = − 1.96, *p* = 0.07). Headache intensity (HIT-6) decreased significantly from 65.7 ± 6.6 at baseline to 59.0 ± 8.4 at 3-months (mean difference − 6.72, 95% CI[− 9.15 to − 4.29], *t* (17) = − 5.84, *p* = 0.01) and disability (WHODAS) was 24.8 ± 16.1 at baseline and 22.0 ± 23.1 at 3-months (mean difference − 2.78 95% CI[− 12.38 to 6.82], *t* (17) = − 0.61, *p* = 0.55) (Fig. 2).

#### Change in neurometabolite levels from baseline to 3-month follow-up

Overall, mean GABA+ levels in the PCG significantly decreased between baseline and 3-month follow-up from 4.93 ± 0.62 IU to 4.48 ± 0.45 IU (mean difference − 0.45 IU, 95% CI [− 0.79 to − 0.10] IU, *t* (17) = − 2.72, *p* = 0.02). At an individual level, a decrease in PCG GABA level was observed in 12 participants, and 6 displayed an increase over the 3-month period. Prior to final analysis, the two outliers (baseline PCG GABA+ 6.5+ and baseline PCG Glx 18+) were reassessed for data quality and modelling and subsequently retained.

**Fig. 3:**
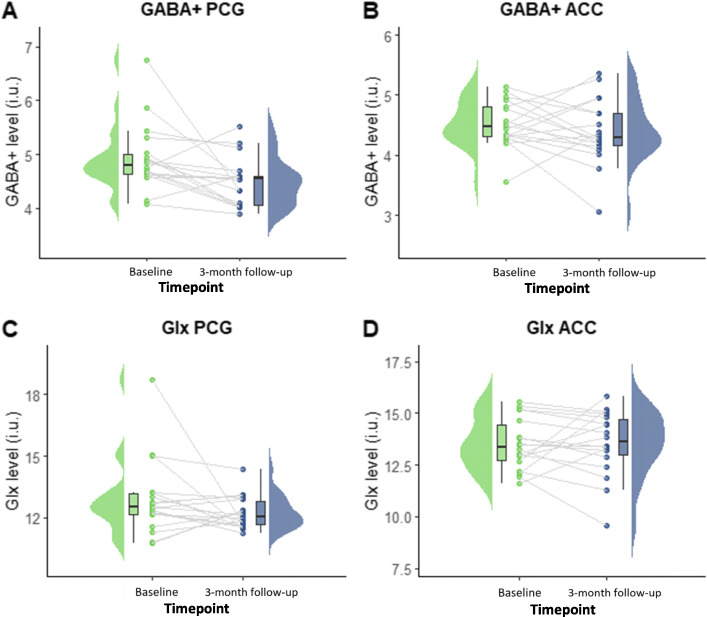
Change in neurometabolite levels between timepoints for participants with migraine. GABA+ in A) PCG, B) ACC, and Glx in C) PCG and D) ACC. Each individual plot represents change in neurometabolite levels from baseline to 3-month follow-up in participants with migraine (*n* = 18). Green data points represent neurometabolite levels at baseline and blue at 3-month follow-up. The grey lines represent change in individual participants neurometabolite levels over time

In contrast to the PCG, mean GABA+ levels in the ACC did not significantly change from baseline to 3-month follow-up. ACC GABA+ levels in participants with migraine at baseline were 4.51 ± 0.38 IU and 3-month follow up 4.40 ± 0.55 IU (mean difference − 0.12 IU, 95% CI [− 0.41 to 0.18] IU, *t* (17) = − 0.85, *p* = 0.41). At an individual level, a decrease in ACC GABA+ levels were observed in 11 participants and an increase in 7. Glx levels were not significantly different between baseline and 3-month follow-up in either the PCG or the ACC (Fig. 3).

### Primary result

#### Correlation between change in brain neurometabolite levels and change in migraine characteristics

**Fig. 4:**
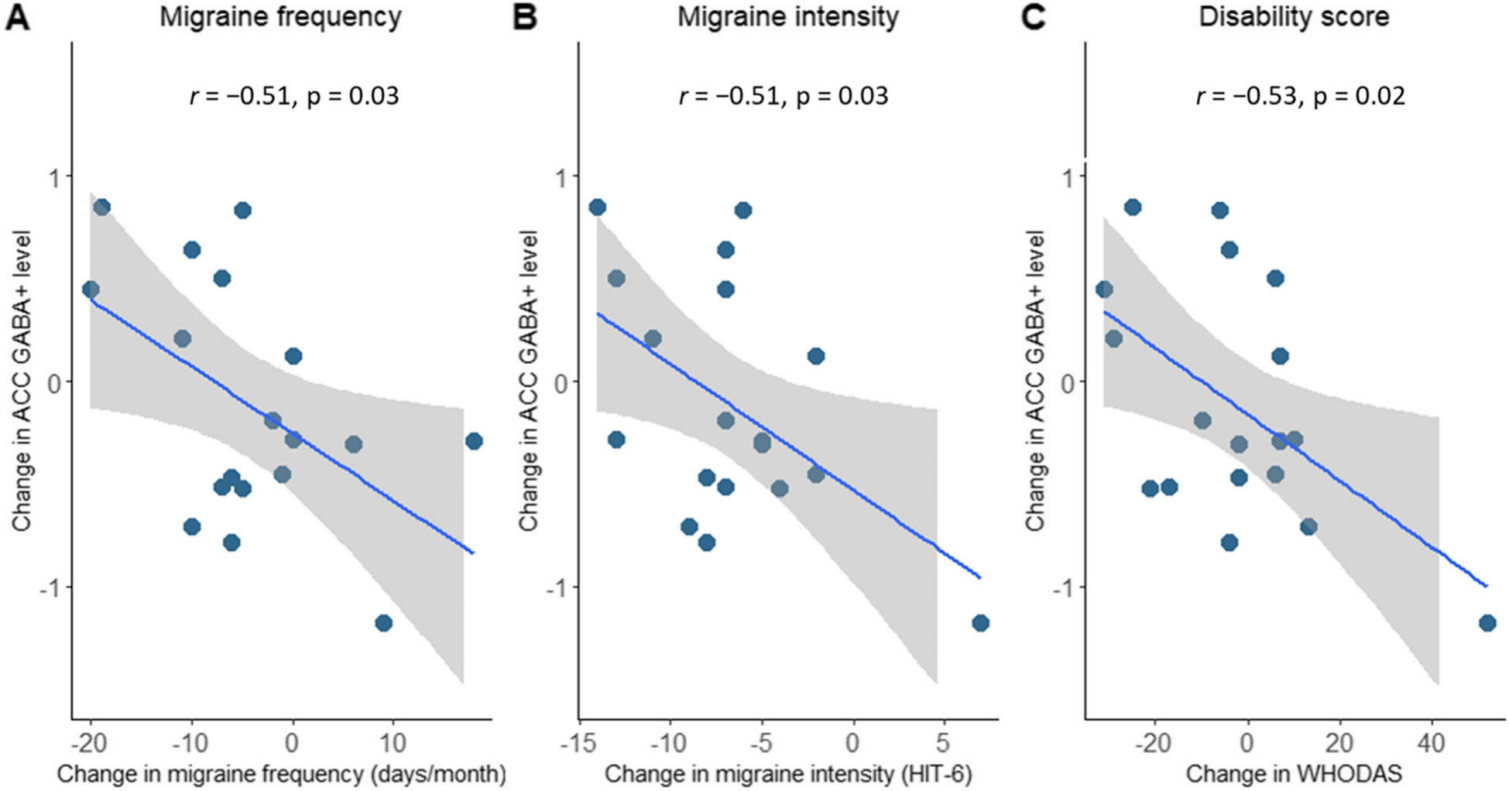
Correlation between change in ACC GABA+ and change in clinical characteristics. Dots represent participants with migraine, the grey ribbon represents the 95% confidence interval, and the blue regression line represents the Pearson’s correlation coefficient *(r)*

There were moderate inverse correlations between GABA+ levels in the ACC and all clinical outcomes. Specifically, we found a moderate inverse correlation between increase in GABA+ levels in the ACC and reduction in headache frequency at 3-month follow-up (*r* = − 0.51, *p* = 0.03 (Table 2, Fig. 4). Similarly, moderate inverse correlations were found between increase in ACC GABA+ levels and both reduction in headache intensity (*r* = − 0.51, *p* = 0.03) and reduction in disability (*r* = − 0.53, *p* = 0.03). In contrast to the findings in the ACC, correlations between change in PCG GABA+ levels and changes in migraine frequency, intensity or disability were negligible and not significant (Table 2). In the case of Glx, there were only negligible correlations between change in Glx and change in clinical characteristics of migraine in both the PCG and the ACC (Table 2, Supplementary materials I).

**Table 2:** Correlation (Pearson’s *r*) between change in GABA+ levels and change in measures of headache frequency, pain intensity and disability in people with migraine (*n* = 18)

Neurometabolite Levels		Change in frequency (days/month) *r* (*p*-value)	Change in intensity (HIT-6) *r* (p-value)	Change in disability (WHODAS) *r* (*p*-value)
Change in GABA+	**PCG**	−0.10 (0.69)	0.01 (0.96)	−0.23 (0.38)
	**ACC**	−0.51 (0.03)*	− 0.51 (0.03)*	− 0.53 (0.02)*
Change in Glx	**PCG**	0.08 (0.74)	−0.07 (0.8)	0.09 (0.74)
	**ACC**	−0.30 (0.23)	−0.32 (0.2)	−0.40 (0.10)

*statistically significant p < 0.05

### Secondary results

#### Correlation between baseline brain neurometabolite levels and change in migraine characteristics

The baseline levels of both GABA+ and Glx in the ACC and PCG demonstrated a negligible correlation with change in migraine frequency, intensity and disability (Supplementary materials I).

#### Correlation between baseline brain neurometabolite levels and change in brain neurometabolite levels

In determining whether baseline neurometabolite levels predict the extent of change in neurometabolite level we found a moderate inverse correlation between baseline levels of GABA+ and change in GABA+ in the ACC (*r* = − 0.54, *p* = 0.01) and strong inverse correlation in the PCG (*r* = − 0.72, *p* = 0.01) respectively. This reflects that those with higher levels of GABA+ at baseline experienced greater reductions in GABA+ over time.

The baseline level of Glx and change in Glx level demonstrated a strong inverse correlation in the PCG (*r* = − 0.92, *p* = 0.01), which was not present in the ACC (*r* = − 0.34, *p* = 0.16).

### Post-hoc analysis

#### Change in GABA+ levels and clinical characteristics in those receiving CGRP-mAbs

Post-hoc analysis demonstrated that those who received CGRP-mAbs (*n* = 10/18) had a significantly greater increase in ACC GABA+ levels and significantly greater improvement in migraine symptoms than those who did not (Supplementary materials II). There was also a moderate positive correlation between receiving CGRP-mAbs (No/Yes) and an increase in ACC GABA+ levels [*r*_pb_ (18) = 0.47, *p* = 0.05] which was not seen in the PCG. Furthermore, there was also a moderate inverse correlation between receiving CGRP-mAbs (No/Yes) and a reduction in migraine frequency [*r*_pb_ (18) = − 0.58, *p* = 0.01] and migraine intensity [*r*_pb_ (18) = − 0.49, *p* = 0.04].

## Discussion

This study sought to measure any changes in GABA+ levels in a group of participants with chronic migraine as their care was escalated. We found that as GABA+ levels increase in the ACC, there was a corresponding moderate correlation with a decrease in migraine frequency, intensity and disability. A chance finding illustrated a greater increase in ACC GABA+ level in those taking CGRP-mAbs. These same correlations were not found in the PCG, despite group mean PCG GABA+ levels changing from baseline to 3-month follow-up. Results from this study suggest that GABA is a key neurometabolite of migraine. Proposed reasons for the differences observed between brain regions are discussed.

A major finding of this study was that an increase in ACC GABA+ levels were associated with an improvement in all three migraine outcomes; namely migraine frequency, intensity, and disability. Furthermore, the associations between change in neurometabolite levels and migraine characteristics were only seen for inhibitory GABA+ but not for excitatory Glx. Together this supports the hypothesis that the balance in cortical excitability in migraine is primarily mediated through inhibitory GABA, rather than excitatory Glx.

To date, correlations between GABA levels and clinical characteristics of migraine have only been measured in cross-sectional studies. Results from these studies have been mixed, with some showing an association with higher GABA/GABA+ levels and higher pain levels [15, 20] and others showing either the opposite [44] or negligible associations [45]. Whilst this discrepancy may be related to methodological differences, the variance in these prior results questions the extent to which GABA+ is related to the clinical characteristics of migraine. A single longitudinal study reported a group mean reduction in PCG GABA+ level in 14 people with migraine following treatment with levetiracetam [46]. Although on average the group in that study improved in terms of both migraine frequency and intensity, the relationship between GABA and clinical characteristics was not explored. Our study examined these associations longitudinally and demonstrated that change in clinical characteristics were associated with change in ACC GABA+ levels. This consequently provides plausible evidence that ACC GABA+ levels are related to pain-related measures of migraine.

An observed correlation between the change in GABA+ levels and change in clinical characteristics of migraine in the ACC but not the PCG is consistent with our understanding of how different brain regions process pain. The role of the ACC in pain processing has been well documented in both pre-clinical and human studies. These studies have demonstrated decreased affective pain behaviour, such as reduced escape behaviour following ACC damage [47], and analgesic responses to direct ACC stimulation [48]. Furthermore, human studies have demonstrated ACC activity during both observing and receiving a painful stimulus [49]. These findings combined with altered ACC GABA+ levels in other pain conditions [22, 23], mean the observed correlation between change in ACC GABA+ and change in clinical characteristics of pain are consistent with the role of the ACC in pain processing.

In contrast, the role of the PCG in pain is less clear. As part of the default mode network, *deactivation* of the PCG has been associated with higher levels of catastrophising in people with migraine [50] and attention to pain in people without a pain condition [51, 52]. Several cross-sectional studies have also demonstrated higher levels of GABA+ in the PCG/visual cortex of people with migraine compared to controls [13–16]. Taken together with the findings of this study, we can posit that GABA+ levels in the PCG might not directly reflect a measure of pain, rather they reflect another aspect of the migraine experience not captured within this study. Consequently, it could be proposed that the ACC provides a more relevant region to explore when investigating the association between GABA+ levels and pain in people with chronic migraine.

Our findings raise the possibility that GABA+ has a pain suppressing role in migraine. Whilst previous cross-sectional reports have identified elevated baseline GABA+ levels in people with migraine compared to pain-free controls, it was not clear if this difference reflected the underlying cause of migraine or an adaptive response to having migraine. Our data support the latter hypothesis suggesting the role of GABA+ is suppressive given that ACC GABA+ further increased as all three clinical measures of migraine reduced. i.e. where ACC GABA+ increased over time, migraine symptoms improved. Further, the reduction in migraine *frequency* may suggest that GABA+ has a role in suppressing cortical sensitivity in migraine, thus increasing the threshold required to trigger a migraine, rather than just modulating the migraine’s severity. This hypothesis suggesting a suppressive role of GABA does not support the proposal that future treatments are required to reduce the elevated GABA+ levels to that observed in healthy participants to better treat migraine [14, 17].

The hypothesised suppressive mechanism of GABA+ in the ACC is further supported by our post-hoc analysis. Although this study is not a drug trial and was not designed to evaluate drug interventions, the use of CGRP-mAbs in 10/18 participants provides a subgroup of people with migraine who experienced greater recovery, e.g. decreased migraine frequency (CGRP-mAbs mean ± SD − 8.8 ± 7.4 days versus Onabotulinumtoxin A 1.5 ± 8.2 days, mean difference 10.3 days, 95% CI [2.52 to 18.07], *p* = 0.01). Accompanying the greater improvement in the CGRP-mAbs group there was also a greater mean increase in ACC GABA+ levels (Supplementary materials II, III). Further, an increase in ACC GABA+ level was observed in 60% (*n* = 6/10) of the CGRP-mAbs group compared to just 12.5% (*n* = 1/8) of the Onabotulinumtoxin A group. This supports the hypothesis that those who improve are more likely to experience an increase in ACC GABA+ levels, providing further evidence that ACC GABA+ levels have a pertinent role in the recovery of people with chronic migraine.

Since both CGRP-mAbs and Onabotulinumtoxin A medications are thought to have a peripheral mode of action and do not cross the blood-brain barrier [53, 54], it is likely that they do not directly influence brain GABA levels. We might speculate that CGRP-mAbs or Onabotulinumtoxin A block the activation of trigeminal afferents by blocking peripheral receptors or inhibiting neuropeptide release [54, 55]. Consequently, tonic or phasic trigeminal afferent drive is inhibited, reducing the activity of neurons in the spinal trigeminal nucleus, thalamus and cingulate cortex pathway [56]. This reduction in afferent drive may ultimately underpin the alteration in excitatory and inhibitory balance observed here in the ACC. In addition, altered descending drive from the cingulate cortex to brainstem pain modulatory circuits, [57, 58] may suppress the ability of trigeminal inputs to evoke a migraine attack. Therefore, it is likely that the correlation between change in ACC GABA+ levels and change in pain levels, reflects the central effects of altered trigeminal afferent drive rather than a direct effect of the medication itself.

### Future directions

This longitudinal study provides the next stage of exploratory research aimed at understanding the role of the neurometabolites GABA and glutamate in migraine. This study reported the composite measures, GABA+ (GABA + macromolecules) and Glx (glutamate-glutamine-glutathione) as they currently represent the most reliable method when using a repeated-measures design [11, 34, 59]. Therefore, some attention should be paid to the macromolecule content of the signal. As technology advances and the specificity and reliability of GABA and glutamate acquisition improve, future studies may wish to use methods that attempt to separate GABA from macromolecules and report glutamate specifically rather than the composite Glx. Further Glx was obtained from the difference spectra. The reliability of this method has been discussed in several studies which suggest although Glx and glutamate can be measured using MEGA-PRESS the measurement of Glx and glutamate may be more reliable if measured using a PRESS sequence [31, 60, 61]. Therefore, our results for Glx would benefit from further investigation using an experiment specifically optimised for Glx.

Further investigation of the temporal nature of GABA+ levels in chronic migraine would aid our understanding. It is hypothesised that the change in GABA+ levels reported in this study might reflect a chronic shift in GABA levels. However, fluctuation of GABA levels in a person with migraine in the short term or throughout the migraine cycle remains unknown. A study of time-resolved measurements, yet to be conducted in a migraine population, may further elucidate the nature of GABA+ changes reported in this study.

The exploratory nature of the study inevitably meant that we were not adequately powered to fully investigate (beyond exploratory testing) subgroups of participants in terms of treatments received or responsiveness. Future research aimed at investigating neurometabolite profiles of people who respond to particular treatments would significantly benefit the migraine community, providing the next step in delivering targeted treatment for migraine. Treatment strategies based on those most likely to respond would not only reduce the unnecessary prescription of medication, but improve patient outcomes, reduce the risk of side-effects, and reduce unnecessary health care costs.

## Conclusion

In conclusion, we found that an increase in ACC GABA+ levels over time was associated with a decrease in migraine frequency, intensity and disability. Suggesting previously reported elevated GABA+ levels may not be a cause of migraine, but a protective mechanism attempting to suppress further migraine attacks. The findings of this study support that ACC GABA may have a pertinent role in the recovery of people with chronic migraine.

## Supplementary Information

**Additional file 1.**

## Data Availability

The open-source software code of Gannet 3.1.3 that was used to process and analyze the MRS data is available from https://github.com/richardedden/Gannet3.1/releases/tag/v3.1.3. The scripts for the batch analysis and the R code for the visualization is available through the OSF repository https://osf.io/y8gps/?view_only=0a35454b80dc456f93e84a99b86fbfb4. Datasets generated during the current study are available from the corresponding authors upon reasonable request.

## Abbreviations

GABA: Gamma-aminobutyric acid
GABA+: GABA + macromolecules
MRS: Magnetic resonance spectroscopy
ICHD: International classification of headache disorders
PBS: Pharmaceutical benefits scheme
HIT-6: Headache intensity scale
WHODAS: World health organization disability assessment schedule
ACC: Anterior cingulate cortex
PCG: Posterior cingulate gyrus
MRI: Magnetic resonance imaging
Glx: Glutamate-gluatmaine-glutathione
CGRP-mAbs: Calcitonin gene-related peptide monoclonal antibodies
