# Supplementary material for: Increase in ACC GABA+ levels correlate with decrease in migraine frequency, intensity and disability over time
Source: J Headache Pain. 2021 Dec 13;22(1):150. doi: 10.1186/s10194-021-01352-1 (PMC8903525; doi:10.1186/s10194-021-01352-1)
Supplement: Supplementary file 1 — Additional file 1. [file 10194_2021_1352_MOESM1_ESM.docx]

**Supplementary materials**

**Supplementary Materials I**

**Table 1: Correlation between baseline GABA+ and clinical characteristics of migraine**

|  | **Change in frequency (days/month)**  *r* (p-value) | **Change in intensity (HIT-6)**  *r* (p-value) | **Change in disability (WHODAS)**  *r* (p-value) |
| --- | --- | --- | --- |
| Baseline GABA+  **PCG**  **ACC** | −0.07 (0.80)  −0.15 (0.57) | −0.17 (0.50)  −0.23 (0.37) | 0.05 (0.85)  0.01 (0.10) |

**statistically significant p <0.05, all negligible correlations*

**Supplementary Materials II**

**Table 2: Post-hoc between group comparison of primary outcomes**

|  | **CGRP-mAbs**  (n = 10) | **Botox®**  (n = 8) | **Mean difference**  [95% CI] | **Independent t-test**  *t* (df), p-value | |
| --- | --- | --- | --- | --- | --- |
| **Change in frequency (days/month)** | −8.8 ± 7.38 | 1.5 ± 8.16 | 10.3 [2.52 to 18.07] | *t* (16) = 2.81, p = 0.01* |  |
| **Change in intensity (HIT-6)** | −8.8 ± 2.97 | −4.13 ± 5.72 | 4.68 [0.26 to 9.09] | *t* (16) = 2.25, p = 0.04* |  |
| **Change in disability (WHODAS)** | −8.4 ± 14.92 | 4.25 ± 22.75 | 12.65 [−6.21 to 31.5] | *t* (16) = 1.42, p = 0.17 |  |
| **Change in ACC GABA+** | 0.12 ± 0.63 | −0.41 ± 0.37 | 0.54 [0.02 to 1.05] | *t* (16) = 2.10, p = 0.05* |  |
| **Change in PCG GABA+** | −0.59 ± 0.82 | −0.27 ± 0.51 | 0.31 [−0.39 to 1.02] | *t* (16) = 0.95, p = 0.36 |  |

*Reported as mean ± SD unless stated, *statistically significant p <0.05*

**9.3 Supplementary Materials III**

**
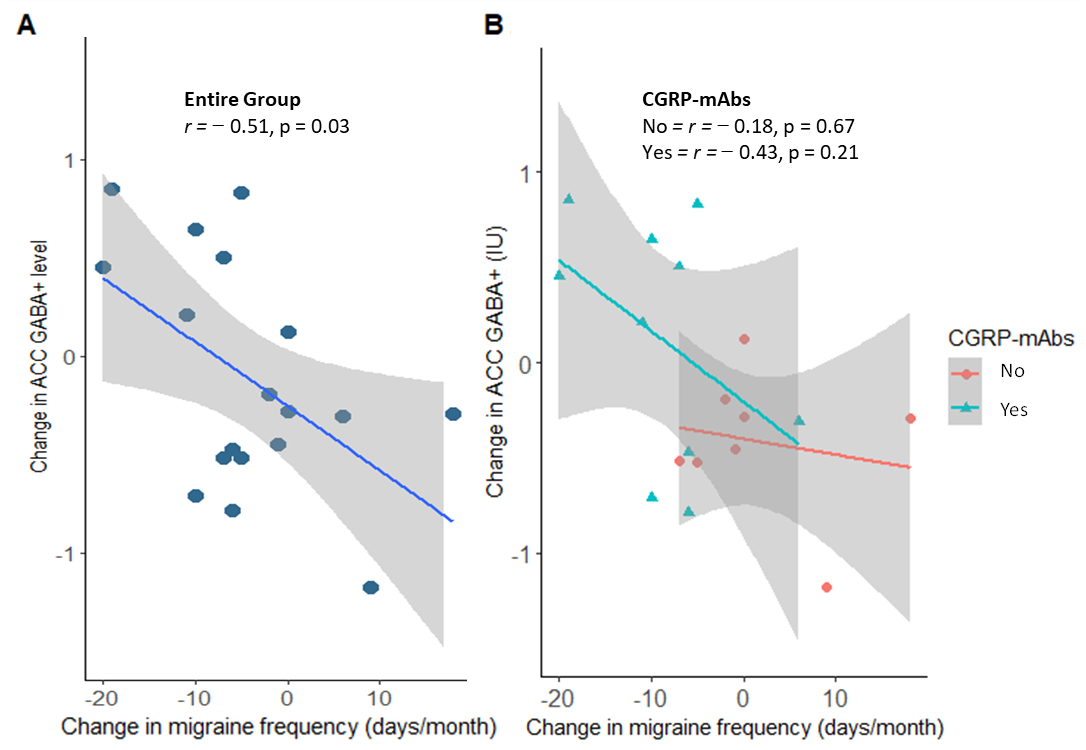
**

**Figure 1: Correlation between change in ACC GABA+ levels and change in migraine frequency.**

Figure 1A) demonstrates the correlation of the whole group of people with migraine reported in this study. Figure 1B) demonstrates the correlation divided by group and shows that the group escalated with CGRP-mAbs had a stronger correlation with an increase in ACC GABA+ than those who did not, although neither correlation reached statistical significance. Further, the people escalated with CGRP-mAbs generally had a greater reduction in migraine days a month compared to those who were not escalated with CGRP-mAbs.
